# Adaptive behaviors in education institutions before and after COVID-19: A systematic literature review

**DOI:** 10.3389/fpsyg.2022.1017321

**Published:** 2022-11-25

**Authors:** Ahmad Walugembe, Joseph Ntayi, Charles Olupot, Joseph Elasu

**Affiliations:** ^1^Faculty of Marketing and International Business, Makerere University Business School, Kampala, Uganda; ^2^Faculty of Economics, Energy and Management Sciences, Makerere University Business School, Kampala, Uganda; ^3^Faculty of Computing and Informatics, Makerere University Business School, Kampala, Uganda

**Keywords:** adaptive work behavior, education institutions, adaptive mechanisms/strategies, adaptive tools, positive adaptive outcomes, COVID-19 pandemic, systematic literature review

## Abstract

**Background:**

The final third of the year 2019 was marked by the outbreak of the deadly coronavirus (COVID-19) pandemic. This virus paralyzed almost all economic sectors, including governments, forcing educational institutions to close. School closures resulted in significant learning losses and increased inequality in the education sector across the world. Despite these disruptions, however, available evidence reveals that some countries quickly developed adaptive mechanisms of emergency remote learning systems and other alternative methods to recover from learning losses, such as televisions, telephones, Zoom, social media, Google Classrooms, email, and even the post office. These learning recovery programs are instrumental in preparing world economies for future shocks. Various studies are being conducted to assess the impact of this pandemic from different sectors and perspectives. However, systematic literature reviews documenting the adaptive behaviors in educational institutions before and after COVID-19 are either sparse or nonexistent. Additionally, systematic reviews provide a synthesis of the available evidence, indicate directions for further research, and inform decision-making. This systematic literature review focuses on documenting the adaptation strategies and subsequent positive outcomes emerging from the COVID-19 pandemic.

**Purpose:**

The purpose of this study was to review published articles on adaptive behavior in educational institutions, and, in particular, review the outcomes of adaptive behavior and coping strategies/mechanisms over time.

**Methodology:**

The study used a systematic literature review approach as a core methodology for defining answerable research questions, searching the literature for the best available evidence, appraising the quality of the evidence, and collecting and aggregating available data for answering the identified questions. The material was collected using the Science Direct and Emerald databases, which are highly regarded as comprehensive and authoritative. Other documents, especially the reports, were collected from the Google Scholar search engine. Search strings used include “COVID-19 adaptive behavior in educational institutions”, “COVID-19 coping strategies in educational institutions”, “COVID-19 adaptation mechanisms in the education sector”, “adaptive work behavior and COVID-19”, “COVID-19 positive outcomes”, “adaptive work behavior in educational institutions”, and “adaptation strategies in educational institutions”.

**Findings and conclusion:**

Although the outbreak of the COVID-19 pandemic wreaked havoc on many aspects of life, the education sector was hit more than most other enterprises, and most, if not all, educational institutions were closed as all students were sent home. Educational institutions transitioned to remote teaching and learning strategies. Several studies report a number of positive outcomes ranging from personal (individuals) and institutional (organizations) to technology-based. Among the key technology-based positive outcomes frequently reported in the literature are increased innovations and increased digital resilience in educational institutions, among others.

## Introduction

The world has been gripped by coronavirus disease (COVID-19) since the first half of 2020, resulting in unprecedented changes in people's way of life (Kniffin et al., 2021). The mitigation strategies, including limiting social contacts, physical distancing, and the temporary shutdown of businesses in all sectors (i.e. lockdowns), had enormous negative economic and health effects (Tusl et al., 2021; Karakose and Malkoc, 2021; Kniffin et al., 2021). As the virus ravaged the world, there was a dramatic increase in unemployment despite the joint efforts of governments and firms to prevent work interruptions (Kniffin et al., 2021). New measures such as “working at home”, teleworking, use of essential workers approach, or rearranging the workspace to maximize physical distancing were implemented (Kniffin et al., 2021).

Previous studies have used the concept of adaptive work behavior with less emphasis on the education sector. For example, a number of studies have been conducted on employee adaptive and nonadaptive outcomes in dynamic work situations (Neal and Griffin, 1999; Pulakos et al., 2000, 2002; Griffin et al., 2007; Bednall and Henricks, 2021), adaptive performance, task proficiency, and proactivity (Griffin et al., 2007) and dimensions of adaptive performance and measurement scales (Charbonnier-Voirin and Roussel, 2012). A number of these studies were also conducted before the outbreak of the coronavirus pandemic in 2019, yet there is also a need to document the adaptive work behavior practices during this period, especially in the education sector given the diverse effects of COVID-19 on the education sector.

Among the highly affected sectors, especially in developing economies, were educational institutions (Mncube et al., 2021). In countries like Uganda, educational institutions were closed for 2 years, and during that period, no normal classes and lectures were organized, and learners were confined to their homes for this period of time (Olum et al., 2020; Twinamasiko et al., 2021). To date, many institutions are facing the challenge of normalizing. In addressing this anomaly, a number of educational institutions had to create different adaptive work behaviors to thrive through the hard times (Karanika-Murray and Biron, 2020; Twinamasiko et al., 2021), and these included virtual learning technologies and tools to facilitate remote learning (Meletiou-mavrotheris et al., 2022), home-schooling (Omara, 2020), radio and televised classes, and school holiday packages (Manyiraho and Atibuni, 2021), among others. Following this trend, the concept of adaptive work behavior has become very important in the education sector, and therefore, understanding its dynamics is of great importance.

Griffin et al. (2010) explained the concept of adaptive work behavior in different spheres, including the degree to which individuals cope with, respond to, and/or support changes resulting in more effective contributions in their roles as individuals, team members, or members of the organization (Griffin et al., 2010), the effectiveness of an individual's response to new demands resulting from the novel and often ill-defined problems created by uncertainty, complexity, and rapid changes in work situations (Schmitt and Chan, 2014), an effective change in response to an altered situation (Dorsey et al., 2017), and employees capability to adapt to rapidly changing work situations (Neal and Griffin, 1999).

Notably, the concept of adaptation is rooted in Darwin's theory of change (Darwin and Kebler, 1859), where issues related to adaptive work behavior and continuous adaptation to changing environmental needs were introduced by Nelson et al. (2007), and since then a number of studies have advanced these concepts in the literature. The American Association on Intellectual and Developmental Disabilities explains adaptive work behavior as the collection of conceptual, social, and practical skills that individuals need to function in their daily lives (Tassé et al., 2012). Martin et al. (2012) indicated that adaptive work behavior is the capacity to modify one's cognition, affect, and behavior constructively, reflecting an individual difference in the way that one responds to changing, new, and uncertain conditions. To summarize, Zhang et al. (2021) posited that adaptability has three main components, i.e., cognitive adjustment, which is the modification of one's thoughts; behavioral adjustment, defined as the modification of one's actions; and effective adjustment, which refers to altering one's effective response (Martin et al., 2012; Zhang et al., 2021).

Adaptive work behavior has been positioned as one of the most relevant change agents in organizations because of its significance to a company's sustainable development, contribution to the company's profitability, long-term continuity, and enhancement of firm competitiveness and performance (Ali et al., 2020). Adaptive work behavior performance can also lead to improved organizational outcomes, including managing change, organizational learning, and keeping up with changing customer expectations (Shoss et al., 2011, 2012). At the individual level, it can facilitate positive outcomes such as enhanced performance capability and career success (Griffin et al., 2007; Shoss et al., 2012) and help employees adapt to change by demonstrating excellence in problem-solving, uncertainty/stress/crisis control, new learning, and adaptability related to people, culture, and environment. Adaptive work behaviors that are necessary for the individual to function well in society may include conceptual, social, and practical skills (Karanika-Murray and Biron, 2020). However, to be adaptive, a person needs to develop cumulative skills to respond to social and community expectations (Karanika-Murray and Biron, 2020).

Studies that have attempted to address the concept of adaptive behavior in the context of educational institutions have focused on a few specific variables like adaptive behavior of students with disabilities (Santos, 2014), adaptive behavior in teaching, adaptive behavior skills for kids with special needs (Godber et al., 2021), adaptive behavior assessment systems, and learning adaptation during COVID-19 (Supena and Hasanah, 2020), impact of COVID-19 on teaching and learning (Kumar et al., 2021), coping strategies, and adaptive behaviors (Orfao et al., 2020), teacher educators as adaptive experts during COVID-19 (Rosehart et al., 2022), influence of COVID-19 confinement on students' performance in higher education (Gonzalez et al., 2020), resilience in higher education (Bento et al., 2021), factors influencing study engagement during the COVID-19 (Koob et al., 2019), and the contribution of COVID-19 to employee negative performance outcomes (Park and Park, 2019; Martin et al., 2020; Raj and Renumol, 2022), among others. These studies did not go further to discuss the positive outcomes emerging from the outbreak of the COVID-19 pandemic. Similarly, although there is evidence of adaptive behavior and coping strategies that existed in educational institutions before the outbreak of COVID-19, the existing literature above have not attempted to document the studies using a systematic literature review approach.

To fill the gaps and increase knowledge in the area of adaptation before and after the COVID-19 period, this study reviews published articles on adaptive behavior in educational institutions. In particular, the study provides a review of existing definitions of adaptive behavior and coping strategies/mechanisms to better understand the topic.

## Materials and methods

A systematic literature review has been developed and used as a core methodology for defining answerable research questions, searching the literature for the best available evidence, appraising the quality of the evidence, and collecting and aggregating available data for answering the identified questions (Petticrew and Roberts, 2008). Unlike traditional literature reviews, systematic literature review methodology employs a transparent but rigorous approach to identify and synthesize all the available research findings of sufficient quality concerning already specified research questions (Higgins and Green, 2008). Although there are biases associated with the systematic literature approach, for example, the use of limiters (time, databases, and journal restrictions), using more than one database makes it possible to capture a wide number of articles to answer the research questions. The steps proposed by Tranfield et al. (2003) include question formulation, locating studies, study selection and evaluation, analysis and evaluation, reporting, and using results.

### Research steps

#### Step 1: Question formulation

To be able to gain an understanding and knowledge of adaptive work behavior, the following questions were formulated to guide the study:

- How is adaptive behavior conceptualized?
- What adaptive mechanisms/strategies were adopted by educational institutions during and after the COVID-19 pandemic?
- What adaptation tools were mostly used in the education sector during and after the COVID-19 pandemic?
- What positive outcomes emerged during and after the COVID-19 pandemic?

#### Step 2: Locating studies

**Figure 1:**
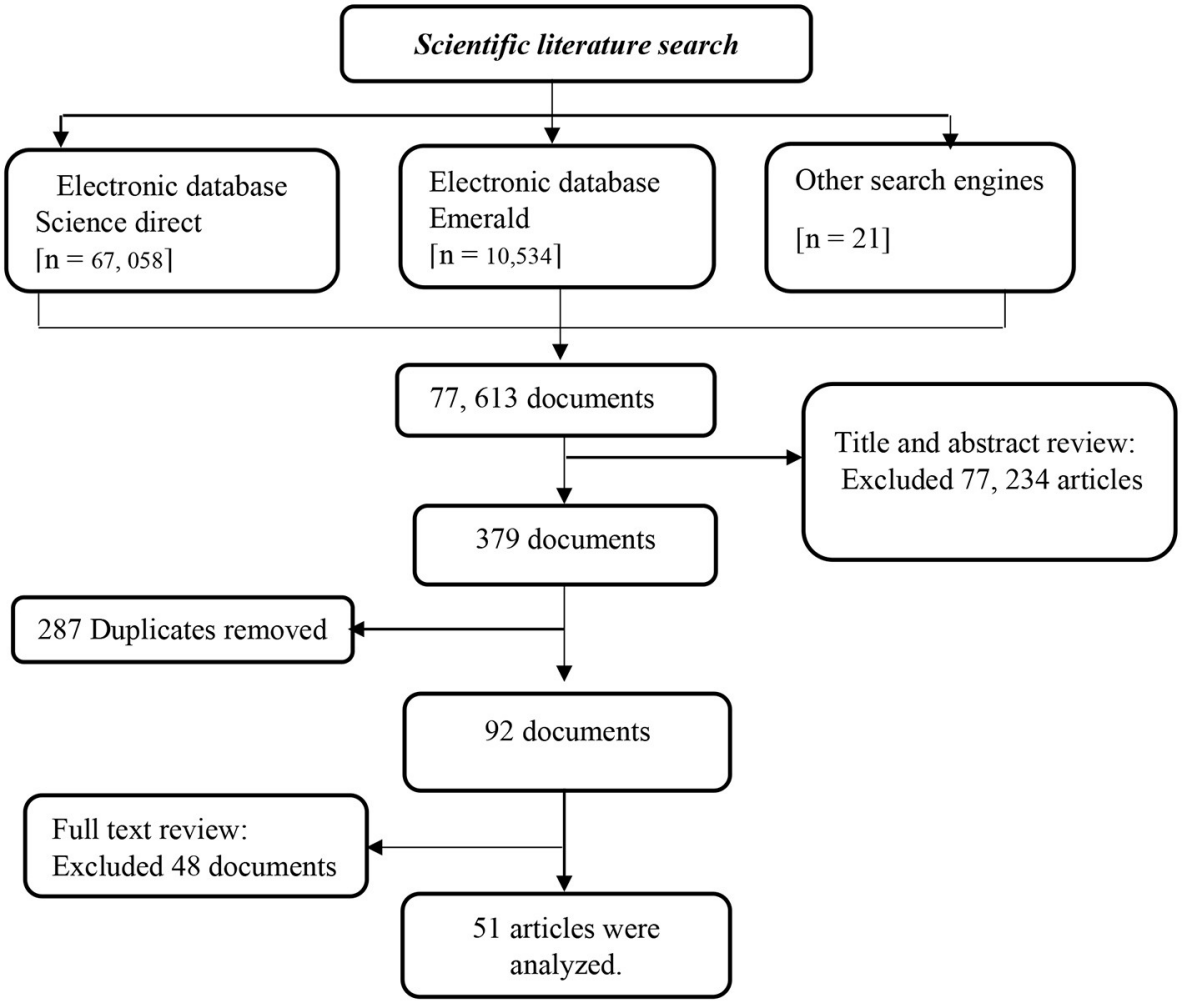
Flowchart for the selection of studies.

The material was collected using Science Direct and Emerald databases, which are highly regarded as comprehensive and authoritative. These databases cover a wide range of peer-reviewed journals, making it easy to access the articles needed for the study. The choice for Emerald and Science Direct was due to the fact that they are the most extensively used databases in literature searches, and most of the bibliometric analysts use these databases for their searches (Aghaei Chadegani et al., 2013; Mongeon and Paul-Hus, 2016). Other search engines, especially Google Scholar, were considered and used during the search process. The databases were searched using search strings or key words (see Table 1) and entire process of article selection is presentation (see Figure 1) in the flow chart.

**Table 1:** Search boundaries and keywords.

**Search boundaries**	**Science Direct, Emerald, Google general, Google Scholar**
**Search terms**	“COVID 19 adaptive behavior in education institutions” “COVID 19 coping strategies in education institutions” “COVID 19 adaptation mechanisms in education sector” “Adaptive work behavior AND COVID 19” “COVID 19 Positive outcomes” “Adaptive work behavior in education institutions” “Adaptation strategies in education institutions”

Regarding journal selection, emphasis was put on the major leading journals. Such journals were identified using the Clarivate (2021) journal ranking and the impact and citation factors. The journals classified and ranked in A, B, and C categories were considered credible journals and were used for content collection. Furthermore, if the Clarivate Analytics classification was not applicable, the journal impact and cite factor were used to identify credible journals from which the articles and records were extracted.

#### Step 3. Study selection and evaluation

In this section, papers were identified and then screened based on the inclusion and exclusion criteria (see Table 2). Given the high volume of papers identified in the first stage, the first exclusion focused on the discipline as indicated in the databases. All papers whose topics were not in line with the main theme of adaptive work behavior were excluded. Papers were further scrutinized alongside the aim of the study. The screening was done using the title and abstract keywords. After the final review and screening, the papers that met the criteria were included in the dataset for analysis.

**Table 2:** Inclusion and exclusion criteria.

**Inclusion**	**Exclusion**
• Peer reviewed journal articles written in English • Articles published between 2000 and 2022. • Research articles aimed at assessing adaptive work behavior, performance/work outcomes are considered.	• Non-peer reviewed journal articles • Literature review articles, • Book chapters, magazines, and conference papers

#### Step 4: Analysis and synthesis

The final step of the analysis summarizes the papers/documents based on the content, type of the study, and field of the research. The full-text articles (records) were analyzed in several steps. Following the steps of Siva et al. (2016), we first established the categories: year, publication, theories, nature of the study, and analysis techniques, as shown in Table 3. The thematic analysis included the conceptualization and adaptation strategies used in different institutions.

**Table 3:** Analytical categories.

**Category**	**Description of the category**
Year	The year in which the article/document was published
Publication	The name of the journal in which the article was published
Analysis technics	Type of analysis techniques used in the study
thematic analysis	Adaptation strategies, tools and positive outcomes emerging from COVID-19

#### Step 5: Reporting

The final step involves reporting the findings of the study and identifying the key research gaps that exist in the literature.

## Analysis and synthesis

This section provides an analysis of reviewed papers along various dimensions. The findings are summarized and presented in tables and figures under different categories.

### Distribution of papers according to the year of publication

**Figure 2:**
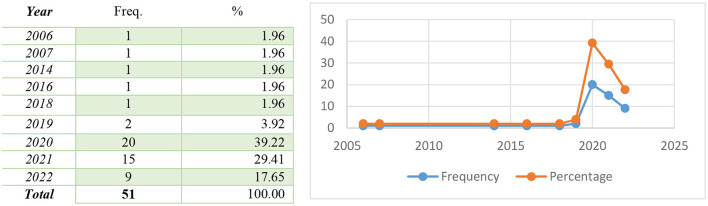
Distribution of the articles overtime.

Figure 2 shows the annual numerical trend for the set of 51 documents reviewed in this study. Findings presented in Figure 2 suggest that a greater percentage (39.2%) of the articles were published in the year 2020.

A good number (29.4%) of the articles were published in 2021, while 17.6% of the articles were published in the year 2022. The remaining 13.8% of the articles were published between 2006 and 2019.

### Journal sources

The 51 documents included in this study were published across 50 different journals, while one document is a report published by United Nations Educational, Scientific and Cultural Organization (UNESCO), United Nations International Children's Emergency Fund (UNICEF), and World Bank. Table 4 shows the list of journals and reputable organizations from which the 51 documents in this study were published. From the table, a significant number of journals have published articles on the study topic. This shows the willingness and interest of different journals in the study area. The findings in the table indicate that *Heliyon, Sustainability*, and *Sustainable Cities and Society* published three (3) documents each, while *Computers in Human Behavior* journal, *Frontiers in Education, Frontiers in Psychology*, and *International Social Work* journal published two articles each. The journals shown in Table 4 contributed at least one article. In this study, gray literature is published by UNESCO, UNICEF, and World Bank.

**Table 4:** Distribution of the articles by journal.

**Publication**	**No. of papers**	**%**
A JOINT UNESCO, UNICEF, AND WORLD BANK REPORT	1	1.96
African Journal of Social Work	1	1.96
Alliance for African Partnership, Michigan State University	1	1.96
Arab World English Journal	1	1.96
Asian Education and Development Studies	1	1.96
BMC Medical Education	1	1.96
Comprehensive Psychiatry journal	1	1.96
Computers in Human Behavior journal	2	3.92
Edtech	1	1.96
Education Research International	1	1.96
Educational Psychology Review	1	1.96
Frontiers in Education	2	3.92
Frontiers in Psychology	2	3.92
Heliyon	3	5.88
Higher Education in the GCC Countries.	1	1.96
Higher Education Studies	1	1.96
Humanities and social sciences communications	1	1.96
Information Systems Management	1	1.96
International Journal of Asian Management	1	1.96
International Journal of Current Research	1	1.96
International Research Journal on Advanced Science Hub	1	1.96
International Social Work journal	2	3.92
Journal of optometry	1	1.96
Journal of Pedagogy, Andragogy and Heutagogy in Academic Practice	1	1.96
Journal of Professional Nursing	1	1.96
Journal of Public Health Research	1	1.96
Journal of Research in Humanities and Social Science	1	1.96
Modern Journal of Language Teaching Methods (MJLTM)	1	1.96
Nurse Education in Practice journal	1	1.96
Pharmacy Education	1	1.96
PLOSE ONE	1	1.96
Policy and Society	1	1.96
Policy Futures in Education	1	1.96
Sage journal	1	1.96
Social Sciences journal	1	1.96
Southern African Journal of Environmental Education	1	1.96
Sustainability	3	5.88
Sustainable Cities and Society	3	5.88
Technology in Society journal	1	1.96
Tertiary Education and Management	1	1.96
The online Journal of Issues in Nursing	1	1.96
**Total**	**51**	100.00

### Distribution of the studies by region

**Figure 3:**
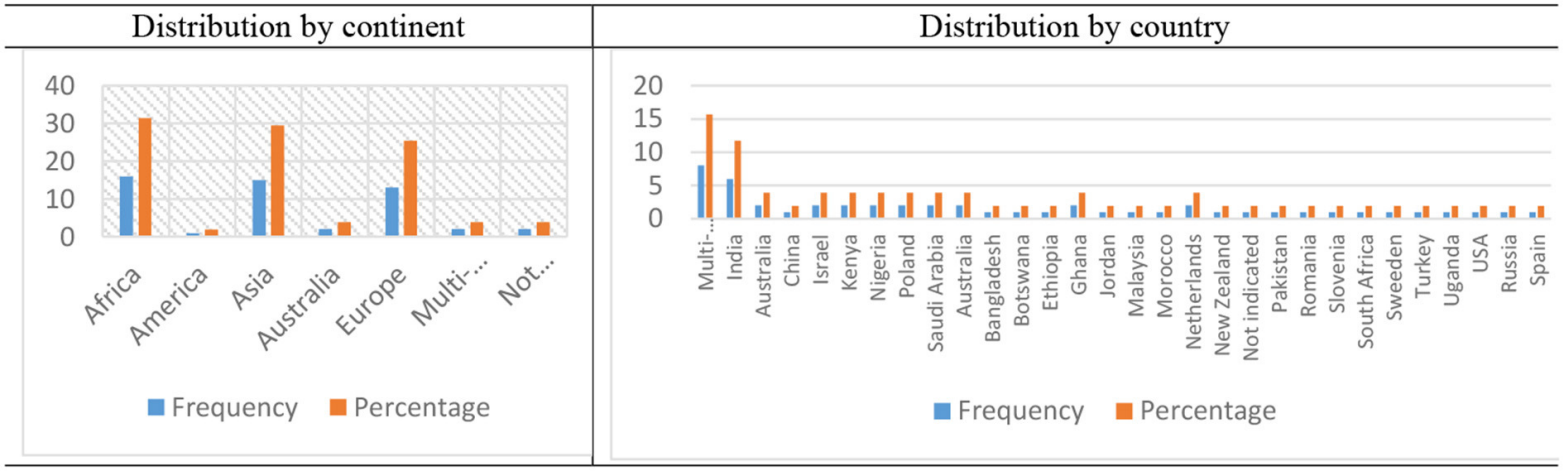
Distribution of the articles by region.

Figure 3 shows the distribution of the articles by region (continent and country). The results show that 15 (31.3%) of the studies were done in Africa. The Asian continent followed closely with 15 (29.4%) studies. A total of 13 studies (25.5%) were carried out in Europe. Findings indicate that the African continent dominated the research on adaptive behavior. It was also found that two studies (3.9%) were conducted in more than one region, while the other 2 studies (3.9%) did not indicate the region from which the study was conducted.

In terms of countries, 8 (15.69%) were conducted in more than one country. China topped the chart with six (11.76%). A significant number of countries (Australia, China, Israel, Kenya, Nigeria, Poland, and Saudi Arabia) published at least two articles included in our dataset. Within the period of study, nineteen countries had at least one article published.

### Data type and data analysis techniques

**Figure 4:**
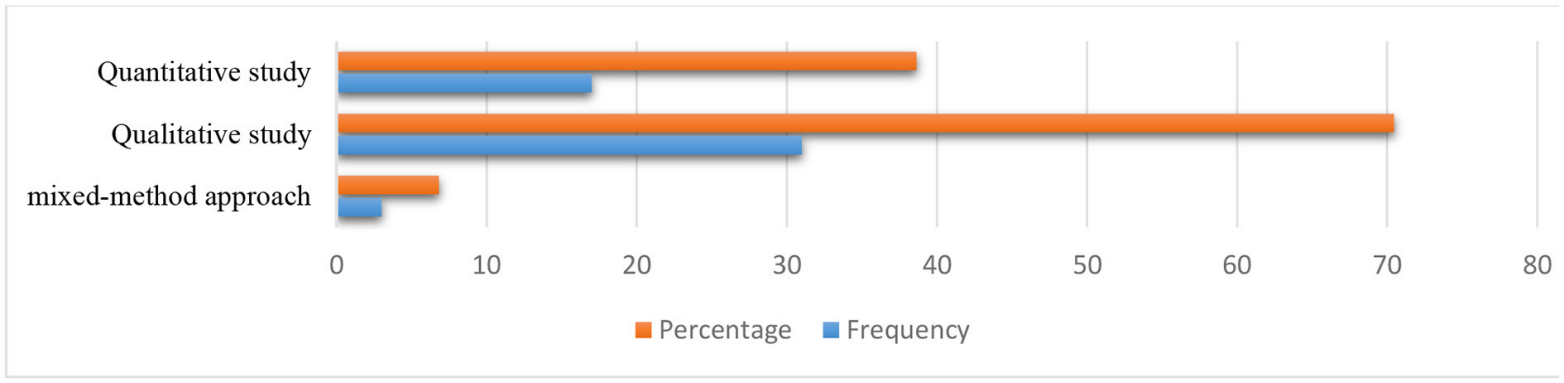
Type of the study.

Figure 4 presents the findings based on the type of study. The findings show that 31 (70.45%) studies were qualitative in nature, and 17 (38.64%) articles were quantitative in nature. A mixed-methods approach was used in three of the studies (6.8%).

Regarding the data analysis tools used (refer to Table 5), descriptive analysis is the most popular data analysis technique (338 papers) (67.9%). This is followed by the Pearson correlation regression technique with three papers (5.4%). Other models used in the papers synthesized included linear regression (2), correlational analysis (3), a k-means cluster analysis (1), ordinal logistic regression analysis (1), meta-analysis (1), inferential analysis (1), and DHW model (1).

**Table 5:** Data analysis tools and techniques.

**Analysis technics**	**Frequency**	**Percentage**
A k-means cluster analysis	1	1.8
ANOVA	1	1.8
Content analysis	1	1.8
Correlational analysis	3	5.4
Descriptive analysis	38	67.9
Inductive content analysis	1	1.8
Inferential analysis	1	1.8
Linear regression	2	3.6
Logistic regression	1	1.8
Metanalysis	1	1.8
Ordinal logistic regression analysis	1	1.8
Pearson correlation regression	3	5.4
RMSEA	1	1.8
SRMR	1	1.8

### Conceptualization of adaptation behavior

Several authors have studied and conceptualized adaption behavior differently, as depicted in Table 6. Although different authors view adaptive behavior differently, they emphasize one's ability to cope with and modify their response to a particular situation with which they are faced (Griffin et al., 2010; Schmitt and Chan, 2014).

**Table 6:** Conceptualization of adaptive behavior.

**Conceptualization**	**Author(s)**
The capacity to modify one's cognition, affect, and behavior constructively reflecting an individual difference in the way that one responds to changing, new, and uncertain conditions.	Martin et al. (2012)
The degree to which individuals cope with, respond to, and/or support changes resulting in more effective contributions in their role as individuals, team members, or members of the organization.	Griffin et al. (2010)
The effectiveness of an individual's response to new demands resulting from the novel and often ill-defined problems created by uncertainty, complexity, and rapid changes in work situations.	Schmitt and Chan (2014)

### Adaptation strategies in educational institutions before the COVID-19 pandemic

Adaptation is the tendency to adjust to the environment (Piaget, 2015). Adaptability is essential for teachers and learners, given the constantly changing demands in the school environment (Bandura, 1977). Table 7 shows different adaptation strategies adopted by both students and teachers in educational institutions before the outbreak of the COVID-19 pandemic. Common among the many adaptation strategies are having regular meetings with supervisors, especially for the students doing higher degrees (18.2%) and actively changing of self (9.1%). Other adaptation behaviors reported in different studies include “active change of environment” and negative “passive subordination to environmental conditions”, increased time of work and commitment, and increased interaction with supervisors and researchers, among others.

**Table 7:** Adaptation behavior before the COVID-19 pandemic.

**Adaptation behavior**	**Freq**.	**%**
Active change of environment	1	4.5
Active change of environment” and negative “Passive subordination to environmental conditions”	1	4.5
Active change of self	2	9.1
Avoiding contact with the environment and immersion in his/her inner world	1	4.5
Practicing emotional and intellectual openness	1	4.5
Freedom in expression of views	1	4.5
Goal setting	1	4.5
Having regular meetings with the supervisor	4	18.2
Increase time of work and commitment,	1	4.5
Increased interaction with supervisors and researchers	1	4.5
Pacing lesson for pupils with Learning Disabilities in regular classroom	1	4.5
Planning	1	4.5
Providing immediate and explicit feedback during lessons	1	4.5
Providing relevant examples during lessons	1	4.5
Adopting self-regulation strategy	1	4.5
Seeking support in daily routine activities	1	4.5
Task analysis	1	4.5
Using positive reinforcement effectively during lessons	1	4.5
**Total**	22	100.0

### Adaptation strategies/mechanisms and tools used during and after the COVID-19 pandemic

Tables 8, 9 show the adaptation strategies/mechanisms adopted by different educational institutions during and after the COVID-19 pandemic. From the findings, education adopted both virtual and non-virtual strategies. The common virtual strategies/mechanisms adopted by educational institutions include virtual online lectures (9), virtual meetings (7), audio conferencing (3), video conferencing (2), and documents posted on the platform (1).

**Table 8:** Strategies adopted by different institutions.

**Virtual learning and teaching strategies**	**Freq**.	**%**
Video conferencing	2	7.4
Virtual meetings	7	11.1
Virtual online lectures	9	14.8
Audio conferencing	3	11.1
Documents posted on the platform	1	3.7
Offering 24 hours of e-library services	1	3.7

**Table 9:** Non-technology adaptation strategies during and after COVID-19.

**Non-technology adaptation strategies**	**Freq**.	**%**
Working from home	4	12.90
Behavioral disengagement	2	6.45
Acceptance of the situation	2	6.45
Active coping	2	6.45
Arranging meetings between learners and their teachers at home	1	3.23
Engaging in physical activities	1	3.23
Using the social values and practices of the indigenous knowledge	2	6.45
Mental Disengagement	2	6.45
Narrative construction and open communication	2	6.45
Practicing yoga and meditation	2	6.45
Promoting awareness	2	6.45
Providing social support	1	3.23
Story telling	2	6.45
Religious coping	2	6.45
Venting	1	3.23
Values and collective responses as the African theory Ubuntu	1	3.23
Positive reframing	1	3.23
Constituting a social network	1	3.23
**Total**	**31**	**100.00**

The common non-virtual adaptation mechanisms employed by educational institutions include working from home (4), behavioral disengagement (2), acceptance of the situation (2), active coping (2), using the social values and practices of the indigenous knowledge (2), mental disengagement (1), narrative construction and open communication (2), practicing yoga and meditation (2), and promoting awareness (2).

The other coping or adaptation strategies adopted during the pandemic, as shown in Table 8, include storytelling (2), religious coping (2), venting (1), values and collective responses such as the African theory of ubuntu (1), positive reframing (1), and constituting a social network (1).

In terms of the tools used by different institutions while coping with the COVID-19 crisis, Table 9 shows that Zoom (27) was the most used, followed by Google Meet (19). Other tools extensively used include Facebook (10), Webex (10), Microsoft Teams (8), WhatsApp (8), Instagram (7), and the Moodle learning app (6).

### Thematic analysis

Table 10 presents the findings from the thematic analysis. Although COVID-19 caused severe negative consequences as documented in the literature, there were also quite a number of positive outcomes that emerged as a result of the pandemic. These positive outcomes were categorized into three themes (individual-based outcomes, organizational-based outcomes, and technology-based outcomes) as shown in Table 11. Under individual-based outcomes, a majority of the studies reported increased autonomy in the learning process (13) as one of the positive outcomes. This was followed by improved personal hygiene (4), digital resilience (4), and students investing more time and effort in their self-study (4). Other positive outcomes under individual-based positive outcomes include enhanced digital literacy (1), improved use of electronic media for sharing information (1), ease and comfort of attending online sessions from home without physically traveling (1), learners are actively searching and bringing the relevant online content to the session (1), being independent of place and time leads to better educational prospects (1), COVID-19 has stimulated stronger activism from learners (1), teachers and students are more engaged than in traditional mode (1), worldwide exposure (interact with peers worldwide wide) (1), and better time management (1).

**Table 10:** Adaptation tools.

**Adaptation tools**	**Freq**	**%**	**Adaptation tools**	**Freq**	**%**
Zoom	27	23.5	BigBlueButton	2	1.7
Google Meet	19	16.5	Email messaging.	2	1.7
Facebook	10	8.7	Skype	2	1.7
Webex	10	8.7	TV lessons,	2	1.7
Microsoft Teams	8	7.0	Twitter	2	1.7
WhatsApp	8	7.0	YouTube	2	1.7
Instagram,	7	6.1	Cisco WebEx	1	0.9
Moodle learning app	6	5.2	Instant messaging app	1	0.9
Cellphone/telephone	3	2.6	LearnCube	1	0.9
The flip classroom	1	0.9	ODeL applications	1	0.9

**Table 11:** Technology-based positive outcomes emerging from the COVID-19 pandemic.

**No**.	**Silver linings emerging from COVID-19**	**Freq**.
	**Individual based outcomes**	
	Enhanced digital literacy	1
	Improved the use of electronic media for sharing information	1
	Ease and comfort of attending online sessions from home without physically traveling	1
	Learners are actively searching and bringing the relevant online content to the session	1
	Being independent of place and time leads to better educational prospects	1
	COVID-19 has stimulated stronger activism from learners	1
	Increased autonomy in learning process	13
	Improved personal hygiene	4
	Students investing more time and effort in their self-study	4
	Teachers and students are more engaged than in traditional mode	1
	Worldwide exposure (interact with peers worldwide wide)	1
	Better time management	1
	**Organizational based outcomes**	
	Teleworking adopted by many organization	1
	Build capacity for technology based learning	1
	Growth of virtual organizations	1
	Development of digital pedagogy	1
	More contextualized responses to education and learning	1
	Roadmap for teachers to teach differently in hard areas while learning digitally	1
	Evolve education systems into something fit for the 21st century	1
	The overcoming of information flow impediments	1
	Fostering of responsible educational activism	1
	Increased university collaboration and partnerships	1
	Development and implementation of online and blended programs	1
	Equal learning opportunity is provided to each students by online teaching	1
	Use of online learning activities facilitate greater engagement of the learner	1
	Improved environmental hygiene (markets and shops**)**	2
	Indigenous knowledge promoted	1
	Improved environmental hygiene	1
	**Technology based outcomes**	
	Rise in use of Learning Management Systems	1
	Increases diffusion of new technologies in education	1
	Increased innovation	10
	Promoted digital resilience	4
	Serve as a catalyst for digitalization of education	1
	Enhanced level of knowledge in technology, diversification in tools used for online Learning, autonomy	1

Under organizational-based outcomes, findings show that improved hygiene (both personal and environmental) was recorded in at least two (2) studies. Other positive outcomes under organizational-based outcomes include the adoption of teleworking by many organizations (1), improved capacity for technology-based learning (1), growth of virtual organizations (1), development of digital pedagogy (1), more contextualized responses to education and learning (1), providing a roadmap for teachers to teach differently in hard areas while learning digitally (1), evolving education systems into something fit for the twenty-first century (1), the overcoming of information flow impediments (1), fostering of responsible educational activism (1), and increased university collaboration and partnerships (1), among others.

The other category (theme) emerging from this study is technology-based outcomes. Under this theme, the most reported positive outcome was an increase in innovation (10). Other positive outcomes emerging from the COVID-19 pandemic under this category include a rise in the use of learning management systems (1), increased diffusion of new technologies in education (1), increased innovation (10), catalyzing the digitalization of education (1), enhanced level of knowledge in technology (1), and diversification in tools used for online learning autonomy (1).

### Affiliated institutions of the authors

Table 12 shows a list of the institutions with which the authors are affiliated. Although Maastricht University (2) and Malmö University (2) had two authors each publishing a paper included in this study, the remaining institutions as shown in Table 12 had one other affiliated with them.

**Table 12:** Affiliated universities/institutions of the authors.

**University/Institutions**	**Freq**.	**University/Institutions**	**Freq**.
Maastricht University	2	Qassim University	1
Malmö University	2	Queensland University	1
Akdeniz University	1	Rhodes University	1
Ashkelon Academic College	1	Rush University	1
Auckland University of Technology	1	Sukkur IBA University	1
Ben-Gurion University of the Negev	1	The American University in Cairo	1
Bielefeld University	1	The Jerzy Kukuczka Academy of Physical Education	1
Bina Nusantara University	1	Transilvania University of Brasov	1
Busitema University	1	Umm al-Qura University	1
Cadi Ayyad University	1	Univerity of Botswana	1
Chouaib Doukkali University	1	Universitat Autònoma de Barcelona	1
Deakin University	1	Universiti Putra Malaysia	1
Delta State University	1	University of Cape Coast	1
Effat University	1	University of Delhi, India	1
Guangdong University of Foreign Studies	1	University of Education Winneba	1
Hebrew University of Jerusalem	1	University of Gdańsk	1
IGNOU Regional Center	1	University of KwaZulu-Nata	1
Imam Abdulrahman Bin Faisal University	1	University of Ljubljana	1
Institute of Management Studies, Mumbai	1	University of Manchester	1
International University of Business Agriculture and Technology	1	University of Melbourne	1
Istanbul Sabahattin Zaim University	1	University of Nairobi	1
JIMMA UNIVERSITY	1	University of Nigeria	1
Jordan University of Science and Technology	1	University of North Texas	1
Kenyatta University	1	University of Oslo	1
Makerere University Business school	1	University of South-Eastern Norway	1
Malegaon Camp Dist. Nashik India	1	University of Southern Queensland,	1
Manipal Academy of Higher Education,	1	University of Stirling	1
Manipal College of Health Professions	1	University of Wollongong	1
MGM School of Biomedical Sciences	1	Victoria University	1
Mohammed VI Polytechnic University	1	Vrije Universiteit Brussel	1
North-West University	1	Zhongnan University of Economics and Law	1
Pomeranian University	1	Prince Sultan University	1

## Discussion

Given the unprecedented consequences of COVID-19, including the deaths of many people within a short period of time, whether there is anything positive emerging out of this pandemic is a crucial question that needs to be answered. In an effort to answer this question, our study attempted to document what researchers have reported as positive outcomes emerging as a result of the COVID-19 pandemic.

This section discusses the positive outcomes that emerged from the outbreak of the COVID-19 pandemic, as presented in Table 11. As demonstrated in the table, three themes (individual-based outcomes, organizational-based outcomes, and technology-based outcomes) were generated, and the subsequent discussion is based on the themes above.

### Technology-based outcomes

This study identified several technology-based outcomes, with a majority of the studies identifying and reporting increased innovations as one of the main positive outcomes that emerged from the COVID-19 outbreak in the world. Educational institutions such as universities are for teaching and learning (Adelowotan, 2021). To ensure the continuity of teaching and learning, educational institutions have deployed a lot of resources to conduct teaching electronically through their websites or learning management systems like Moodle. This innovative technology has enabled learners to interact using online modes of teaching and learning (Adelowotan, 2021).

The development of digital pedagogy became the contingency plan to achieve teaching and learning during the COVID-19 crisis. The advantage is that even after the crisis, digital pedagogy has continued to operate in educational institutions (Zhang and Yu, 2021). It is crucial to note that the education sector worldwide is undergoing a technological reform using hybrid teaching and learning featuring productivity, flexibility, and connectivity guided by different technological applications (Ng et al., 2017; Zhang and Yu, 2021). Researchers (Anderson, 2020; Zhang and Yu, 2021) are in agreement that the increased adoption of technology in education is a strong ingredient in augmenting teaching and learning. A pedagogy using technology has the potential to build resilience to future educational challenges (Anderson, 2020; Zhang and Yu, 2021). Digital and educational philosophy changed with emerging technologies. This has helped in connecting formal institutions with wider communities that transcend social and regional differences. The advantage of digital pedagogy is the promotion of learner-centered instruction and a flexible learning environment that minimizes burnout among learners and their instructors (Teräs et al., 2020).

Other than the growth in innovations in line with teaching and learning, COVID-19 created a fertile breeding ground for novel solutions and approaches (Ramalingam and Prabhu, 2020). Universities have been involved in developing COVID-19 test kits (Ramalingam and Prabhu, 2020). For example, Makerere University's department of immunology and molecular biology, during the time of the pandemic, developed COVID-19 Rapid Antibody Test Kits. This innovation was a response to the high cost of using imported items. This outcome was a result of the emergence of the COVID-19 pandemic (Diaz et al., 2021; Sahito et al., 2022).

While many educational institutions worked independently to develop their own innovations, the outbreak of COVID-19 drove a number of educational institutions, especially universities, into multi-university collaboration and partnerships. In choosing to collaborate during the crisis, educational institutions were able to demonstrate that they can work together and create innovations and lasting solutions to the problems facing the world. As a result, educational institutions not only generate valuable innovations to sustain education and improve it but also contributed to transforming internal processes in a way that enhanced their own ability to fulfill the mandate placed upon many of the universities to serve as research institutions (Ramalingam and Prabhu, 2020).

### Organizational-based positive outcomes

Along with other important behaviors such as physical distancing, hygiene became one of the key positive outcomes that resulted from the COVID-19 crisis. Several studies (Gebru, 2020; Alghamdi and Id, 2021; Hagan et al., 2022) confirm that an improvement in hygiene has been observed among students and within the environment as a result of the COVID-19 pandemic. Outside of educational institutions, scholars (Alghamdi and Id, 2021) further argue that the banning of social gatherings such as wedding parties led to a positive impact on the environment and hygiene. Moreover, the closing of shopping malls and implementation of curfews reduced the amount of waste generated from commercial centers, thus improving the environment and hygiene.

Improvement in social connectedness stems as one of the major positive outcomes of the COVID-19 pandemic among both students and their parents. Although the lockdown measures denied people movement to other places and instead confined them at home, it is important to note that people were able to bond with one another as a result of being confined at home for a long period of time (Alghamdi and Id, 2021). In this case, the pandemic acted as a catalyst for feelings of social unity, strengthening the connectedness of families and the entire society (Alghamdi and Id, 2021). Continued interaction as all family members were confined at home helped everyone appreciate life and death, recognize priorities in life, and create a feeling of social unity (Dhawan, 2020). The pandemic also forced students to develop certain skills such as problem-solving, critical thinking, and, most importantly, adaptability to survive the crisis (Dhawan, 2020).

### Individual-based positive outcomes

Several individual-based positive outcomes have been reported in the literature. Increased autonomy in the learning process is one such positive individual-based outcome frequently reported in the literature (Naus et al., 2015; Lusinga and de Groot, 2019; Woodruff, 2019; Ransan-Cooper et al., 2020; Biwer et al., 2021; Bögel et al., 2021). As COVID-19 ravaged the world and locked most, if not all, educational institutions, students were introduced to a system that promoted autonomy, where the learners were required to study on their own. Learner autonomy requires that a student take control and responsibility for his or her own learning, both in terms of what he/she learns and how to learn it. The beauty in this is that the student becomes independent and can develop proactive approaches to his/her studies.

Another positive individual-based positive outcome emerging from the COVID-19 pandemic was an enhancement of digital literacy in both instructors and learners (Lusinga and de Groot, 2019; Middleton et al., 2019; Sankhyayan and Dasgupta, 2019). Although a number of universities were using online platforms even before COVID-19, when lockdowns were instituted, most, if not all, educational institutions had to close, and that meant that for learning to continue, both teachers and learners had to adapt to the online system to ensure continuity of teaching and learning. This, in a way, enabled the enhancement of digital skills for both the learners and teachers. It should also be noted that in the process of enhancing digital skills, learners also found it not only easy to attend lectures at home without physically attending class but also with active involvement in searching relevant content online, they benefited more as compared to the traditional ways of learning. Increased digital skills have also been widely recognized as one of the positive outcomes arising from the outbreak of the pandemic (Jena, 2020). Teachers and learners have the opportunity to interact with peers around the world, and as a result, learners get adapted to the international community (Jena, 2020). This was made possible because educational institutions built the capacity for technology-based learning with diverse tools that enhance online learning (Diaz et al., 2021; Sahito et al., 2022).

## Concluding remarks

### Conclusion

Although the outbreak of the COVID-19 pandemic wreaked havoc on many aspects of life, the education sector was significantly impacted by the pandemic, and most, if not all, educational institutions were closed and their students sent to their respective homes. Educational institutions transitioned to remote teaching and learning strategies. With the pandemic's glaring negative consequences, it became difficult to imagine any positive outcomes emerging from this pandemic. This systematic literature study sought to document some of the positive outcomes reported in the literature as a result of the COVID-19 pandemic. Several studies report a number of positive outcomes ranging from personal (individuals), and institutional (organizations) to technology-based outcomes. Key among the technology-based positive outcomes frequently reported in the literature include increased innovations and increased digital resilience in educational institutions. It is therefore necessary to insist that although digital innovations existed in educational institutions even before the outbreak of the COVID-19 pandemic, there was an unprecedented level of increase in innovations with the emergence of COVID-19, resulting in positive adaptations among students and teachers.

### Limitations

There are a few limitations to this study. First, there is bias in the selection of databases, papers, and publications. This means that the papers found in other databases that were not consulted during the search were left out. Second, the bias in the search strings meant that all publications that were without the search strings could not be collected and evaluated for this study. Finally, the bias from rejecting all the papers published in languages other than English. Useful publications not in the English language were left out even though they seemed to contain the content the researchers were interested in.

To ensure that the above biases were controlled, the authors ensured that, other than the database, other search engines, including Google, were consulted to collect the publications that could have been missed from the main databases used for data collection. The authors also ensured that the keywords/search strings were developed based on the objectives of the study and that the inclusion and exclusion procedures were planned in such a way that the objectives of the study were met.

### Feature research

Our study focused on the adaptation behavior and work outcomes in educational institutions before, during, and after the COVID-19 pandemic. Our findings show that educational institutions responded swiftly by adopting adaptation strategies and mechanisms to ensure the continuity of teaching and learning. We went further to profile the tools that were adopted by educational institutions before, during, and after the COVID-19 pandemic. Adopting these tools, mechanisms, and strategies is one thing, but the successful use of such technologies is another thing. Future studies should be done to document how successful were these strategies and what the rate of academic outcomes compared with the traditional methods of instruction. Furthermore, scholars could consider doing similar studies in other sectors and profile the adaption and behavioral work outcomes before, during, and after the COVID-19 pandemic.

## Data availability statement

The raw data supporting the conclusions of this article will be made available by the authors, without undue reservation.

## Author contributions

All authors listed have made a substantial, direct, and intellectual contribution to the work and approved it for publication.

## Conflict of interest

The authors declare that the research was conducted in the absence of any commercial or financial relationships that could be construed as a potential conflict of interest.

## Publisher's note

All claims expressed in this article are solely those of the authors and do not necessarily represent those of their affiliated organizations, or those of the publisher, the editors and the reviewers. Any product that may be evaluated in this article, or claim that may be made by its manufacturer, is not guaranteed or endorsed by the publisher.

## References

[B1] Adelowotan M. (2021). Educational innovations for coping up with covid-19 situation in south african universities^*^. Eur. J. Educ. Res. 95, 139–155. 10.14689/ejer.2021.95.8

[B2] Aghaei Chadegani A. Salehi H. Md Yunus M. M. Farhadi H. Fooladi M. Farhadi M. . (2013). A comparison between two main academic literature collections: Web of science and scopus databases. Asian Soc. Sci. 9, 18–26. 10.5539/ass.v9n5p18

[B3] Alghamdi A. A. Id A. A. A. (2021). Impact of the COVID-19 pandemic on the social and educational aspects of Saudi university students' lives. PLoS ONE 16, 1–18. 10.1371/journal.pone.0250026 33852627 PMC8046245

[B4] Ali S. A. Yassin M. AbuRaya R. (2020). The impact of firm characteristics on corporate financial performance in emerging markets: Evidence from egypt. Int. J. Custom. Relat. Market. Manage. 11, 70–89. 10.4018/IJCRMM.2020100105

[B5] Anderson V. (2020). A digital pedagogy pivot: re-thinking higher education practice from an HRD perspective. Human Resour. Dev. Int. 23, 452–467. 10.1080/13678868.2020.1778999

[B6] Bandura A. (1977). Self-efficacy: Toward a unifying theory of behavioral change. Psychol. Rev. 84, 191–215. 10.1037/0033-295X.84.2.191 847061

[B7] Bednall T. C. Henricks M. D. (2021). “Adaptive performance,” in Global Perspectives on Change Management and Leadership in the Post-COVID-19 Era, 71–89.

[B8] Bento F. Giglio Bottino A. Cerchiareto Pereira F. Forastieri de Almeida J. Gomes Rodrigues F. (2021). Resilience in higher education: a complex perspective to lecturers' adaptive processes in response to the covid-19 pandemic. Educ. Sci. 11, 492. 10.3390/educsci11090492

[B9] Biwer F. Wiradhany W. Oude Egbrink M. Hospers H. Wasenitz S. Jansen W. . (2021). Changes and adaptations: how university students self-regulate their online learning during the COVID-19 pandemic. Front. Psychol. 12, 1–12. 10.3389/fpsyg.2021.642593 33967903 PMC8103204

[B10] Bögel P. M. Upham P. Shahrokni H. Kordas O. (2021). What is needed for citizen-centered urban energy transitions: Insights on attitudes towards decentralized energy storage. Energy Policy 149, 112032. 10.1016/j.enpol.2020.112032

[B11] Charbonnier-Voirin A. Roussel P. (2012). Adaptive performance: a new scale to measure individual performance in organisations. Can. J. Admin. Sci. 29, 280–293. 10.1002/cjas.232

[B12] Clarivate (2021). Journal Citation Reports 2021 Release (2020 Data) (Issue July).

[B13] Darwin C. Kebler L. (1859). On the Origin of Species by Means of Natural Selection, or, The Preservation of Favoured Races in the Struggle for Life. 30164232 PMC5184128

[B14] Dhawan S. (2020). Online learning: a panacea in the time of COVID-19 crisis. J. Educ. Technol. Syst. 49, 5–22. 10.1177/004723952093401833935298

[B15] Diaz K. Staffileno B. A. Hamilton R. (2021). Nursing student experiences in turmoil: a year of the pandemic and social strife during final clinical rotations. J. Prof. Nurs. 37, 978–984. 10.1016/j.profnurs.2021.07.019 34742531 PMC8564681

[B16] Dorsey D. W. Cortina J. M. Allen M. T. Waters S. D. Green J. P. Luchman J. . (2017). “Adaptive and citizenship-related behaviors at work,” in Handbook of Employee Selection, Second Edition, 448–475.

[B17] Gebru A. (2020). Psychosocial impacts of COVID-19 lockdown and coping strategies of the community, Jimma University, Southwest Ethiopia. Afr. J. Soc. Work 10, 41–49.

[B18] Godber K. A. Atkins D. R. Bailey C. (2021). COVID-19 impacts on teaching and learning: A 26 collaborative autoethnography by two higher education lecturers. Front. Educ. 6, 1–14. 10.3389/feduc.2021.647524

[B19] Gonzalez T. De la Rubia M. A. Hincz K. P. Comas-Lopez M. Subirats L. Fort S. . (2020). Influence of COVID-19 confinement on students' performance in higher education. PLoS ONE 15, 1–23. 10.1371/journal.pone.0239490 33035228 PMC7546684

[B20] Griffin M. A. Neal A. Parker S. K. (2007). A new model of work role performance: positive behavior in uncertain and interdependent contexts. Acad. Manage. J. 50, 327–347. 10.5465/amj.2007.24634438

[B21] Griffin M. A. Parker S. K. Mason C. M. (2010). Leader Vision and the Development of Adaptive and Proactive Performance: A Longitudinal Study. J. Applied Psychology 95, 174–182. 10.1037/a0017263 20085414

[B22] Hagan J. E. Quansah F. Ankomah F. Agormedah E. K. Srem-Sai M. Frimpong J. B. . (2022). Linking COVID-19-related awareness and anxiety as determinants of coping strategies' utilization among senior high school teachers in cape Coast Metropolis, Ghana. Soc. Sci. 11, 137. 10.3390/socsci11030137

[B23] Higgins J. P. H. Green S. (2008). “Cochrane handbook for systematic reviews of interventions,” in Proceedings of the IEEE International Symposium on Information Theory.35352103

[B24] Jena D. P. K. (2020). Impact of pandemic COVID-19 on education in India. Int. J. Curr. Res. 12, 12582–12586. 10.31235/osf.io/2kasu

[B25] Karakose T. Malkoc N. (2021). Psychological impact of the COVID-19 pandemic on medical doctors in Turkey. Social Behavior and Personality: An International Journal. 49, e9890.

[B26] Karanika-Murray M. Biron C. (2020). The health-performance framework of presenteeism: Towards understanding an adaptive behaviour. Human Relations 73, 242–261. 10.1177/0018726719827081

[B27] Kniffin K. M. Narayanan J. Anseel F. Antonakis J. Ashford S. P. Bakker A. B. . (2021). COVID-19 and the workplace: implications, issues, and insights for future research and action. Am. Psychol. 76, 63–77. 10.1037/amp0000716 32772537

[B28] Koob C. Schröpfer K. Coenen M. Kus S. Schmidt N. (2019). Factors influencing study engagement during the COVID 19 pandemic: a cross—sectional study among health and social professional students. PloS ONE. 16, e0255191. 10.1371/journal.pone.0255191 34314450 PMC8315536

[B29] Kumar A. Sarkar M. Davis E. Morphet J. Maloney S. Ilic D. . (2021). Impact of the COVID-19 pandemic on teaching and learning in health professional education: a mixed methods study protocol. BMC Med. Educ. 21, 1–7. 10.1186/s12909-021-02871-w 34412603 PMC8374410

[B30] Lusinga S. de Groot J. (2019). Energy consumption behaviours of children in low-income communities: a case study of Khayelitsha, South Africa. Energy Res. Soc. Sci. 54, 199–210. 10.1016/j.erss.2019.04.007

[B31] Manyiraho D. Atibuni D. Z. (2021). Adoption of technology enhanced teaching and learning innovations during Covid-19 lockdown in rural Uganda. 2021 IST-Africa Conference, IST-Africa 2021, 1–10.

[B32] Martin A. J. Nejad H. Colmar S. Liem G. A. D. (2012). Adaptability: conceptual and empirical perspectives on responses to change, novelty and uncertainty. J. Psychol. Counsell. Schools 22, 58–81. 10.1017/jgc.2012.8.0

[B33] Martin F. Dennen V. P. Bonk C. J. (2020). A synthesis of systematic review research on emerging learning environments and technologies. Educ. Technol. Res. Dev. 68, 1613–1633. 10.1007/s11423-020-09812-2 32837122 PMC7377308

[B34] Meletiou-mavrotheris M. Eteokleous N. Stylianou-georgiou A. (2022). education sciences Emergency Remote Learning in Higher Education in Cyprus during COVID-19 Lockdown : a zoom-out view of challenges and opportunities for quality online learning. Educ. Sci. 12, 477. 10.3390/educsci12070477

[B35] Middleton L. Hall H. Raeside R. (2019). Applications and applicability of social cognitive theory in information science research. J. Librarianship Inf. Sci. 51, 927–937. 10.1177/0961000618769985

[B36] Mncube V. S. Mutongoza B. H. Olawale B. E. (2021). Managing higher education institutions in the context of COVID-19 stringency: experiences of stakeholders at a rural south african university. Perspect. Educ. 39, 390–409. 10.18820/2519593X/pie.v39.i1.24

[B37] Mongeon P. Paul-Hus A. (2016). The journal coverage of Web of Science and Scopus: a comparative analysis. Scientometrics 106, 213–228. 10.1007/s11192-015-1765-5

[B38] Naus J. Van Vliet B. J. M. Hendriksen A. (2015). Households as change agents in a Dutch smart energy transition: on power, privacy and participation. Energy Res Soc Sci. 9, 125–136. 10.1016/j.erss.2015.08.025

[B39] Neal A. Griffin M. A. (1999). Developing a model of individual performance for human resource management. Asia Pac. J. Human Resour. 37, 44–59. 10.1177/103841119903700205

[B40] Nelson D. R. Adger W. N. Brown K. (2007). Adaptation to environmental change: contributions of a resilience framework. Ann. Rev. Environ. Resour. 32, 395–419. 10.1146/annurev.energy.32.051807.090348

[B41] Ng J. L. Collins C. E. Knothe Tate M. L. (2017). Engineering mechanical gradients in next generation biomaterials – Lessons learned from medical textile design. Acta Biomaterialia 56, 14–24. 10.1016/j.actbio.2017.03.004 28274765

[B42] Olum R. Atulinda L. Kigozi E. Nassozi D. R. Mulekwa A. Bongomin F. . (2020). Medical Education and E-Learning During COVID-19 Pandemic: Awareness, Attitudes, Preferences, and Barriers Among Undergraduate Medicine and Nursing Students at Makerere University, Uganda. J. Med. Educ. Curr. Dev. 7, 238212052097321. 10.1177/2382120520973212 33283049 PMC7682244

[B43] Omara P. (2020). Learning at home during covid-19 pandemic in abim district, uganda: learners' perspectives. J. Educ. Soc. Sci. 16, 139–146.

[B44] Orfao N. H. Ferreira M. de Souza G. Feitosa V. G. Martins L. M. (2020). COVID-19: coping strategies and adaptive behaviors adopted by health professionals during the pandemic. Revista De Epidemiologia E Controle De Infeccao 10, 100–500. 10.17058/reci.v10i4.15462

[B45] Park S. Park S. (2019). Employee adaptive performance and its antecedents: review and synthesis. Human Resour. Dev. Rev. 18, 294–324. 10.1177/1534484319836315

[B46] Petticrew M. Roberts H. (2008). “Systematic reviews in the social sciences: a practical guide,” in Systematic Reviews in the Social Sciences: A Practical Guide (New York, NY: Wiley).

[B47] Piaget J. (2015), in Encyclopedia of Educational Theory and Philosophy, eds D. C. Phillips. (2014). Newcastle upon Tyne, United Kingdom: Sage.

[B48] Pulakos E. D. Arad S. Donovan M. A. Plamondon K. E. (2000). Adaptability in the workplace: development of a taxonomy of adaptive performance. J. Appl. Psychol. 85, 612–624. 10.1037/0021-9010.85.4.612 10948805

[B49] Pulakos E. D. Schmitt N. Dorsey D. W. Arad S. Hedge J. W. Borman W. C. (2002). Predicting adaptive performance: Further tests of a model of adaptability. Human Perform. 15, 299–323. 10.1207/S15327043HUP1504_01

[B50] Raj N. S. Renumol V. G. (2022). A systematic literature review on adaptive content recommenders in personalized learning environments from 2015 to 2020. J. Comput. Educ. 9. 10.1007/s40692-021-00199-4

[B51] Ramalingam B. Prabhu J. (2020). “Innovation, development and COVID-19 : challenges, opportunities and ways forward,” in OECD Tackling Coronavirus (COVID-19): Contributing to a Global Effort, 2–30.

[B52] Ransan-Cooper H. Lovell H. Watson P. Harwood A. Hann V. (2020). Frustration, confusion and excitement: Mixed emotional responses to new household solar-battery systems in Australia. Energy Res. Soc. Sci. 70, 101656. 10.1016/j.erss.2020.101656

[B53] Rosehart P. Hill C. Sivia A. Sadhra S. St. Helene J. (2022). Seeking serendipity: teacher educators as adaptive experts during COVID. J. Educ. Teach. 00, 1–15. 10.1080/02607476.2022.2082275

[B54] Sahito Z. Shah S. S. Pelser A. Page A. (2022). Online Teaching during COVID-19 : exploration of challenges and their coping strategies faced by university teachers in Pakistan. Front. Educ. 7, 1–12. 10.3389/feduc.2022.880335

[B55] Sankhyayan P. Dasgupta S. (2019). ‘Availability' and/or ‘Affordability':What matters in household energy access in India? Energy Policy 131, 131–143. 10.1016/j.enpol.2019.04.019

[B56] Santos S. (2014). Adaptive behaviour on the Portuguese Curricula: A Comparison between Children and adolescents with and without intellectual disability. Creat. Educ. 10.4236/ce.2014.57059

[B57] Schmitt N. Chan D. (2014). Adapting to Rapid Changes at Work: Definitions, Measures and Research. London, UK: Routledge.

[B58] Shoss M. K. Witt L. A. Vera D. (2012). When does adaptive performance lead to higher task performance? J. Organ. Behav. 33, 910–924. 10.1002/job.780

[B59] Shoss M. K. Witt L. A. Vera D. (2011). When does adaptive performance lead to higher task performance? J. Marriage Family 60, 5–22.

[B60] Siva V. Gremyr I. Bergquist B. Garvare R. Zobel T. (2016). The support of Quality Management to sustainable development: a literature review. J. Clean. Prod. 138:148–157. 10.1016/j.jclepro.2016.01.020

[B61] Supena A. Hasanah U. (2020). Teaching models for children with moderate intellectual disabilities during Covid-19 pandemic. Lentera Pendidikan: Jurnal Ilmu Tarbiyah Dan Keguruan 23, 295. 10.24252/lp.2020v23n2i9

[B62] Tassé M. J. Schalock R. L. Balboni G. Bersani H. Borthwick-Duffy S. A. Spreat S. . (2012). The construct of adaptive behavior: Its conceptualization, measurement, and use in the field of intellectual disability. Am. J. Intell. Dev. Disabil. 117, 291–303. 10.1352/1944-7558-117.4.291 22809075

[B63] Teräs M. Suoranta J. Teräs H. Curcher M. (2020). Post-Covid-19 Education and Education Technology ‘Solutionism': a Seller' s Market Content courtesy of Springer Nature, terms of use. Postdigit Sci Educ, 2. 10.1007/s42438-020-00164-x PMC7355515 40477107

[B64] Tranfield D. Denyer D. Smart P. (2003). Towards a Methodology for Developing Evidence-Informed Management Knowledge by Means of Systematic Review ^*^. BJM 14, 207–222. 10.1111/1467-8551.00375

[B65] Tusl M. Kerksieck P. Brauchli R. Bauer G. F. (2021). Perceived impact of the COVID-19 crisis on work and private life and its association with mental well-being and self-rated health in german and swiss employees: A cross-sectional study. BMC Health. 21, 741. 10.1186/s12889-021-10788-8 33865354 PMC8052554

[B66] Twinamasiko N. Nuwagaba J. Maria Gwokyalya A. Nakityo I. Wasswa E. Sserunjogi E. . (2021). Drivers affecting the acceptance and use of electronic learning among Ugandan University students in the COVID-19 era: a cross-sectional survey among three universities. SAGE Open 11. 10.1177/21582440211029922

[B67] Woodruff J. N. (2019). Accounting for complexity in medical education: a model of adaptive behaviour in medicine. Med. Educ. 53, 861–873. 10.1111/medu.13905 31106901

[B68] Zhang J. Yu S. (2021). Reconceptualising digital pedagogy during the COVID-19 pandemic: A qualitative inquiry into distance teaching in China. Innov. Educ. Teach. Int. 00, 1–11. 10.1080/14703297.2021.2000473

[B69] Zhang K. Wu S. Xu Y. Cao W. Goetz T. Parks-Stamm E. J. . (2021). Adaptability promotes student engagement under COVID-19: the multiple mediating effects of academic emotion. Front. Psychol. 11, 1–8. 10.3389/fpsyg.2020.633265 33488491 PMC7815756

